# Maqui berry extract prevents cigarette smoke induced oxidative stress in human osteoblasts *in vitro*

**DOI:** 10.17179/excli2020-3244

**Published:** 2021-02-09

**Authors:** Sheng Zhu, Romina H. Aspera-Werz, Tao Chen, Weidong Weng, Bianca Braun, Tina Histing, Andreas K. Nüssler

**Affiliations:** 1 Department of Traumatology, Eberhard Karls University Tübingen, BG Clinic, Siegfried Weller Institute, Schnarrenbergstraße 95, 72076 Tübingen, Germany

**Keywords:** cigarette smoke, Maqui berry extract, human osteoblasts, oxidative stress, Nrf2

## Abstract

Oxidative stress which can be induced by cigarette smoke (CS) is associated with an altered osteoblast differentiation, and an inhibition of the mineralization process. Therefore, treatments focusing on reducing oxidative stress in osteoblasts could be a potential therapy supporting bone formation. Maqui berry extract (MBE) is the richest natural source of delphinidins with high antioxidant activity. In the present study, we pre-/ co-/ post-incubated MBE in cigarette smoke extract (CSE)-affected human osteoblasts (hOBs), to investigate the effects of MBE as an antioxidant on hOBs. Our results clearly showed that high concentrations of MBE are toxic for hOBs, while physiological concentrations of MBE have no negative effects *in vitro*. Physiological concentrations of MBE can reduce oxidative stress caused by CSE in hOBs by activating the antioxidative regulator Nrf2 and its regulated antioxidative enzymes. Moreover, the physiological concentration of MBE prevents the detrimental effects of CSE-induced oxidative damage on hOBs by increasing cell viability, differentiation capability and matrix mineralization. Pre-incubation with MBE showed a positive effect on the activation of the cellular antioxidant system in hOBs. Thus, we conclude that MBE at physiological concentrations can effectively protect osteoblasts from oxidative stress-induced damage by activating the cells' antioxidative defense system.

## Introduction

Cigarette smoking (CS) is the single leading and preventable cause of death worldwide (Ding et al., 2019[8]). CS is associated with many systemic diseases (Lee et al., 2018[25]; Onor et al., 2017[38]) and has been regarded as a major risk factor that triggers the development of bone diseases like osteoporosis (Fusaro et al., 2018[17]). However, the underlying mechanism of the detrimental effects of CS on bone metabolism is still not completely elucidated. In terms of current research results, bone formation is tightly affected by reactive oxygen species (ROS) which may inhibit osteoblastic function (Domazetovic et al., 2017[9]). Increased oxidative stress is associated with the inhibition of osteoblast differentiation and the mineralization process (Romagnoli et al., 2013[43]). Previous studies of our lab clearly showed that mesenchymal stem/stromal cells exposed to cigarette smoke extract (CSE) lead to increased oxidative stress by formation of superoxide radicals, which then negatively affects differentiation process and bone formation (Aspera-Werz et al., 2018[3]; Sreekumar et al., 2018[46]). Consistent with our results, CSE demonstrated a damaging effect on osteoblast in both *in vitro* (Braun et al., 2011[4]) and *in vivo* (Reumann et al., 2020[42]) models. Antioxidants could counteract these negative effects of oxidative stress, favoring an improved osteoblastic activity and viability, and maintain a normal bone remodeling process (Domazetovic et al., 2017[9]; Wauquier et al., 2009[51]). Therefore, treatments focused on preventing oxidative stress in osteoblasts could be a potential therapy supporting bone formation or at least preventing further damage in smokers.

As a crucial role in resistance to oxidant stress, the nuclear factor erythroid 2-related factor 2 (Nrf2) is substantially associated with a broad range of cellular toxicity and diseases related to oxidative pathology (Klaassen and Reisman, 2010[22]; Ma and He, 2012[30]). Nrf2 directly regulates intercellular antioxidant defense systems through a multitude of mechanisms to modulate the homeostasis of ROS (Li et al., 2019[26]; Ma, 2013[29]). In particular, the transcription of cytoprotective genes of major antioxidant enzymes such as superoxide dismutase (SOD), catalase (CAT), and glutathione peroxidase (GPX) are promoted by activated Nrf2 (Qi and Tang, 2020[39]). Ultimately, the Nrf2-regulated antioxidant system ensures cells to respond to oxidants adequately in time and space (Elekofehinti et al., 2020[16]).

ROS-targeted therapeutic interventions with naturally occurring anthocyanins are becoming one of the ultimate treatments against devastating illnesses (Ullah et al., 2019[49]). Anthocyanins from different natural plants could inhibit oxidative stress by activating Nrf2 expression, and antioxidant enzymes (Liu et al., 2020[28]; Molagoda et al., 2020[34]). Among all the anthocyanin species, delphinidins have shown the most potent antioxidative properties (Watson and Schonlau, 2015[50]). Specifically, Delphinol® Maqui berry extract (MBE) from which is standardized to 25 % delphinidin, is the richest natural source of delphinidins up to now with high antioxidant capacity (Ruiz et al., 2010[44]; Watson and Schonlau, 2015[50]). Recently, the Maqui berry has been considered to possess a wide range of beneficial nutritional and health-promoting features due to its anti-oxidative properties, including preventing cardiovascular diseases, diabetes and cancers (Masoodi et al., 2019[33]). 

Regarding bone health, MBE was shown in an osteopenic mouse model to potentially prevent bone loss by stimulating bone formation and inhibiting bone resorption (Nagaoka et al., 2019[36]). Moreover, anthocyanin-rich extract administration can ameliorate bone loss induced by diabetes or RANKL activation (Nagaoka et al., 2019[37]; Qi et al., 2019[40]). Nevertheless, more detailed studies are needed to fully understand how Maqui berry and its active components interact with human physiological and pathological processes of skeletal system.

In the present study, we exposed primary human osteoblasts (hOBs) to CSE and MBE to investigate the effects of MBE as an antioxidant on untreated or CSE-affected hOBs. 

## Materials and Methods

### Ethics statement

Human studies were accomplished according to the 1964 Declaration of Helsinki in its latest amendment. The study protocol was approved by the ethics committee of the Medical Faculty of the Eberhard-Karls-Universität and University clinic Tübingen (ethical vote: 538/2016BO2). All participants completed a written consent form in the study. Tissues from patients with (potential) tumor or infectious diseases were excluded from this study.

### hOBs isolation and cultivation

Medical consultation was mandatory before harvesting bone tissue. All bone samples were obtained from the Department of Traumatology, BG Unfallklinik Tübingen. Bone fragments were isolated from cancellous bone samples and digested with 0.07 % w/v Collagenase II (Serva, Heidelberg, Germany) in PBS for 1 h. Released hOBs were cultured in Dulbecco's Modified Eagle's Medium (DMEM)-high glucose 5 % v/v fetal calf serum (FCS), 50 µmol/L L-ascorbate-2-phosphate, 100 U/mL penicillin and 100 µg/mL streptomycin, 50 µmol/L β-glycerol-phosphate after washing with PBS. Medium change was performed every 4 days. Culture medium was replaced by osteogenic medium (DMEM-high glucose, 1 % v/v FCS, 5 mM β-glycerol-phosphate, 200 µM L-ascorbate-2-phosphate, 1.5 mM CaCl_2_, 25 mM HEPES, and 100 nmol/L dexamethasone) to induce osteogenic differentiation (Ehnert et al., 2018[14]).

### Preparation of MBE

Delphinol^®^ is a standardized MBE product that consists of a minimum of 25 % delphinidins, which has a high antioxidant capacity (Gillespie et al., 2007[18]). Delphinol^®^ is a highly water-soluble powder extract and therefore was dissolved in cell culture medium (without FCS) and diluted to different concentrations (Nagaoka et al., 2019[36]). MBE was sterilized by a 0.22 μm pore filter and then used in the cell cultures.

### Preparation of CSE

One commercial cigarette (Marlboro, Philip Morris, New York City, USA) was continuously bubbled through a 25 mL pre-warmed plain DMEM-high glucose medium without FCS in a standard gas wash bottle. A peristaltic pump was used to generate negative pressure at a speed of 95-100 bubbles/ min. The CSE was normalized using a plate reader (BMG Labtech, Ortenberg, Germany) by the optical density at 320 nm (OD_320_). 0.7 of OD_320_ was considered as 100 % CSE. The normalized CSE was diluted with osteogenic differentiation medium after sterile filtration (0.22 μm pore filter) into 5 % CSE, which is associated with smoking 10 cigarettes/day (Sreekumar et al., 2018[46]).

### Experimental set-up

**Figure 1:**
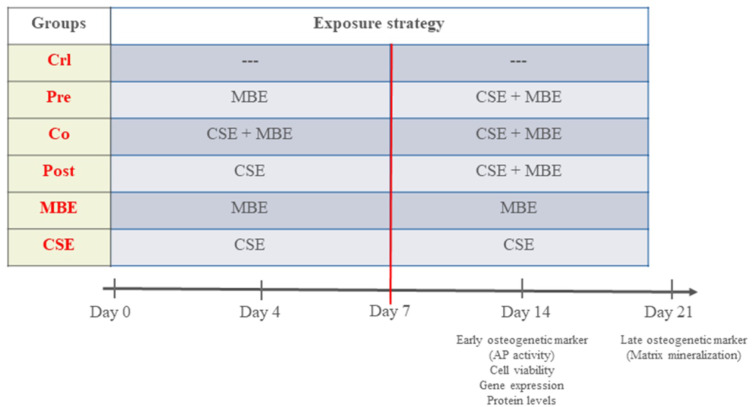
The experimental set-up in the study MBE= Maqui berry extract; CSE= Cigarette smoke extract

To evaluate the effects of CSE and MBE, and their combined effects on hOBs, the present study was grouped as follows: Control group (Crl): hOBs were cultured in osteogenic medium without supplement up to 21 days; Pre-incubation group (Pre): hOBs were cultured in osteogenic medium with MBE for 7 days and CSE was added subsequently till day 21; Co-incubation group (Co): hOBs were exposed to CSE and MBE in osteogenic medium for 21 days; Post-incubation group (Post): hOBs were cultured in osteogenic medium with CSE for 7 days, and MBE was added subsequently till day 21; Maqui group (MBE): hOBs were incubated with MBE in osteogenic medium for 21 days; CSE group (CSE): hOBs were incubated with CSE in osteogenic medium for 21 days. Osteogenic medium was changed twice a week, and hOBs were exposed to CSE and MBE by changing 10 % volume of medium every day to maintain a certain concentration (Figure 1(Fig. 1)). Measurements were performed on day 4, 7 and 14 for AP activity, cell viability assays, and analysis of gene and protein expression, and on day 21 for the matrix mineralization measurement (late osteogenic marker).

### Resazurin conversion assay

Mitochondrial activity of cells was detected by Resazurin conversion assay. Cells were incubated with 0.025 % w/v Resazurin solution (Sigma-Aldrich, Darmstadt, Germany) (1:10 diluted in PBS) for 30 min at 37 °C. Formed resorufin was measured by fluorescence test (ex/em = 540/590 nm) using a plate reader (BMG Labtech, Ortenberg, Germany), and Resazurin solution without incubation with cells was considered as the background control (solvent mixture without cells) (Ehnert et al., 2016[11]).

### Sulforhodamine B (SRB) staining

SRB staining is a measurement for total protein content of cells. Cells were directly fixed by ethanol for at least 60 min at −20 °C after washing with PBS. After fixation, cells were incubated with 0.4 % w/v SRB for 30 min at room temperature protected from light. Unbound SRB was removed by washing with 1 % v/v acetic acid, and bound SRB was resolved with 10 mmol/ L unbuffered TRIS solution (pH = 10.5). SRB staining results were determined by absorbance test (λ = 565 nm) with a plate reader (Aspera-Werz et al., 2018[3]).

### Calcein AM/ Hoechst staining

Living cells were determined by calcein AM (intracellular esterase activity) and nucleus were determined by Hoechst 33,342 staining. hOBs were incubated with 2 µM calcein AM and 3.5 µM Hoechst 33,342 (diluted in PBS) at 37 °C for 30 min after washing with PBS. A fluorescence microscope (Epifluorescence: EVOS FL, life technologies, Darmstadt, Germany) was used to detect living cells (green) and cell nucleus (blue).

### ROS level determination

2′,7′-dichlorofluorescein-diacetate (DCFH-DA) as a fluorescent dye can reveal ROS production in cells. hOBs were incubated with 10 µM DCFH-DA for 30 min at 37 °C. After washing with PBS twice, cells were directly exposed to different concentrations of MBE or 5 % CSE. hOBs which were exposed to 0.001 % v/v H_2_O_2 _were considered as the positive control. The increase in fluorescence was detected by a plate reader (ex/em=  485/520 nm) (Chen et al., 2020[6]).

### Alizarin red staining

Matrix mineralization, which is mediated by the well-differentiated osteoblasts, was measured by Alizarin red staining. Cells were washed with PBS and then directly fixed with ethanol at − 20 °C for at least 60 min. After fixation, cells were washed by using tap water three times and incubated with Alizarin red solution (0.5 % w/v, pH = 4.0) for 30 min at room temperature. Stained matrix (red) can be detected by microscope after washing with tap water at this stage. The bound staining was resolved with 10 %w/v cetylpyridinium chloride solution, and the quantification of alizarin staining was determined by measuring the absorbance (λ = 562 nm) using a plate reader (Ehnert et al., 2015[12]).

### Alkaline Phosphatase (AP) activity

AP activity was correlated to early osteoblastic differentiation. Cells were washed with PBS and incubated with AP reaction solution (100 mM TRIS, 0.2 % 4-nitrophenyl-phosphate, 50 mM glycine, 1 mM MgCl_2_, pH = 10.5) at 37 °C for 15 min. AP activity results were measured photometrically (λ = 405 nm), and AP reaction solution without incubation with cells was used for control. Results were normalized to cell numbers of hOBs determined by SRB staining (Ehnert et al., 2017[15] (85 V for 40 - 50 min) was carried out to separate the gel).

### Gene expression analysis

PCR measurements were used to determine the gene expression. Trifast reagent (38 % v/v phenol, 0.4 mM ammonium thiocyanate, 0.8 mM guanidine thiocyanate, 0.68 mM glycerol, and 3 M sodium acetate solution) was used to isolate total RNA, and quantified using a plate reader. cDNA synthesis was performed by using the cDNA kit according to the manufacturer (Thermo Fisher Scientific, Massachusetts, the USA). Biozym Red HS Taq Master Mix (Vienna, Austria) was used to perform PCR reactions by the guide of manufacturer's manual. The PCR conditions and primer sequences for the investigated genes are shown in Table 1(Tab. 1). *18S* was used for housekeeping gene. RNA samples were loaded onto a 1.8 % w/v agarose gel with ethidium bromide. Gel electrophoresis (85 V for 40 - 50 min) was carried out to separate the gel (Ehnert et al., 2017[13]). As a reference for the amplification size of DNA, pUC19/ Msp I (Carl Roth, Karlsruhe, Germany) was used as a DNA marker. Intensity of the bands was measured with ImageJ software (NIH, Bethesda, USA).

### Protein level analysis

Cell lysates of hOBs were prepared by using RIPA buffer, and micro Lowry assay was used to quantify total protein of hOBs. Cell lysates were diluted to obtain a protein content of 1 ng/ μL in ddH_2_O. 20 ng protein was transferred to a nitrocellulose membrane via a Dot blotter (Carl Roth, Karlsruhe, Germany). Ponceau staining was performed to confirm the transfer of proteins. Membranes were washed with Tris-buffered saline with Tween 20 (TBS-T) to remove ponceau and then blocked with 5 % BSA in TBS-T for 1 h at ambient temperature. Membranes were incubated with primary antibodies (see detailed information in Table 2(Tab. 2)) overnight at + 4 °C, and GAPDH was used for the housekeeper. The next day membranes were incubated with the secondary antibodies for 2 h at ambient temperature after washing with TBS-T. ECL substrate solution was used for chemiluminescent signal development of membranes. Signals of proteins were detected by a ChemCam imager (INTAS, Göttingen, Germany), and the intensity of signals was quantified by using the ImageJ software (Weng et al., 2020[52]).

### Statistics

GraphPad Prism Software 8.0 (El Camino Real, USA) was used for statistical calculations and figure plotting in the study. Results are obtained from experiments that have been repeated at least three times and are expressed as mean ± SEM. The number of biological (N) and technical (n) replicates is shown in the figure legends. Data sets were compared by non-parametric Kruskal Wallis test followed by Dunn's multiple comparison test using *p* < 0.05 was taken as minimum level of significance.

## Results

### Effects of MBE on hOBs

**Figure 2:**
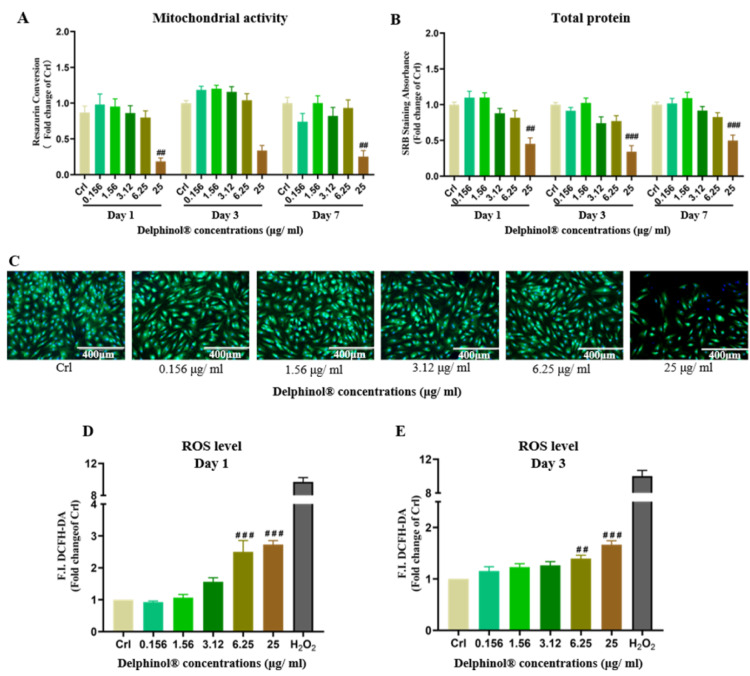
Effects of MBE on hOBs. (A) Measurement of the resazurin conversion of hOBs exposed to different concentrations of MBE on day 1, day 3, and day 7 of culture. (B) SRB staining of hOBs exposed to different concentrations of MBE on day 1, day 3, and day 7 of culture. Data are represented as mean ± SEM. Significance is determined as ^#^*p* < 0.05, and ^##^*p* < 0.001 vs. Crl group. (C) Representative Calcein AM/Hoechst 33,342 staining microscopy images of hOBs exposed to different concentrations of MBE for 7 days. Red arrows indicate that hOBs had the highest cell viability when exposed to 1.56 μg/ml Delphinol^®^. (D) & (E). ROS level measurements of hOBs exposed to different concentrations of MBE on day 1 and 3 of culture. Data are represented as mean ± SEM, and the significance is determined as ^##^*p* < 0.01, and ^###^*p* < 0.001 vs. Crl group.

We determined cytotoxicity of MBE on hOBs and its ability to induce oxidative stress with concentrations of MBE ranging from 0 - 25 μg/mL. MBE was shown to be toxic to hOBs at concentrations ≥ 25 μg/mL, decreasing resazurin conversion and SRB staining significantly. on day 1, 3 and 7 (Figure 2A and 2B(Fig. 2)). Calcein AM/Hoechst staining also showed that treatment with 25 μg/mL MBE resulted in a significant reduction in the viability of hOBs on day 7 (Figure 2C(Fig. 2)). Additionally, we found high concentrations of MBE significantly increased ROS levels in hOBs (Figure 2D and 2E(Fig. 2)). Low concentrations of MBE had no negative effect on hOBs cell viability and did not increase the ROS level. Among the tested concentrations, 1.56 μg/mL MBE demonstrated the highest hOBs cell viability, which is consistent with the physiological concentrations in humans (Schon et al., 2018[45]). Based on this result, a concentration of 1.56 μg/mL MBE was determined to be used in subsequent experiments. 

### Incubation with MBE decreases oxidative stress induced by CSE exposure

**Figure 3:**
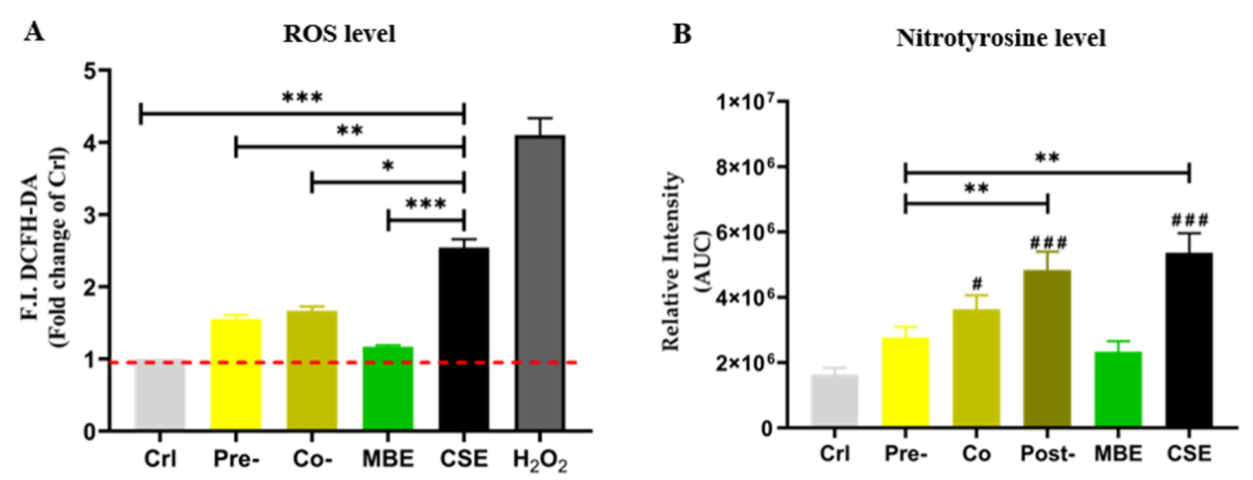
The oxidative stress level of hOBs under different exposure strategies of CSE and MBE. (A) In MBE and CSE group, hOBs were incubated with MBE and CSE respectively for 30 min, and in Co-group hOBs were incubated with CSE and MBE simultaneously for 30 min. In Pre-group hOBs were pre-incubated with MBE for 4 h, and afterwards were incubated with 5 % CSE for the next 30 min, H_2_O_2_ group was used for positive control. Data are represented as the mean ± SEM, and the significance was represented as *p < 0.05, **p < 0.01, and ***p < 0.001 vs. CSE group. (B) Nitrotyrosine levels of hOBs were detected by Dot blot method. Area under the curve (AUC) between day 4 to day 14 of nitrotyrosine levels is shown. Data are represented as mean ± SEM, significance is represented as ***p < 0.001. and ^#^*p* <0.05, ^##^*p *<0.01, and ^###^*p* <0.001 vs. Crl group (N = 3, n ≥ 3).

Nitrotyrosine is a ROS-mediated tyrosine nitration product, which can be used to indicate the level of oxidative stress in cells (Knight et al., 2018[23]). Therefore, ROS levels and nitrotyrosine levels were measured to determine the oxidative stress in the present study. As shown in Figure 3(Fig. 3), both ROS and nitrotyrosine levels were increased significantly by CSE in hOBs. Incubation with MBE could significantly decrease the levels of ROS and nitrotyrosine induced by CSE. Pre-incubation with MBE had the most pronounced effect in reducing elevated nitrotyrosine by CSE.

### MBE and CSE exposure activate Nrf2 in hOBs, and incubation with MBE could further activate Nrf2

**Figure 4:**
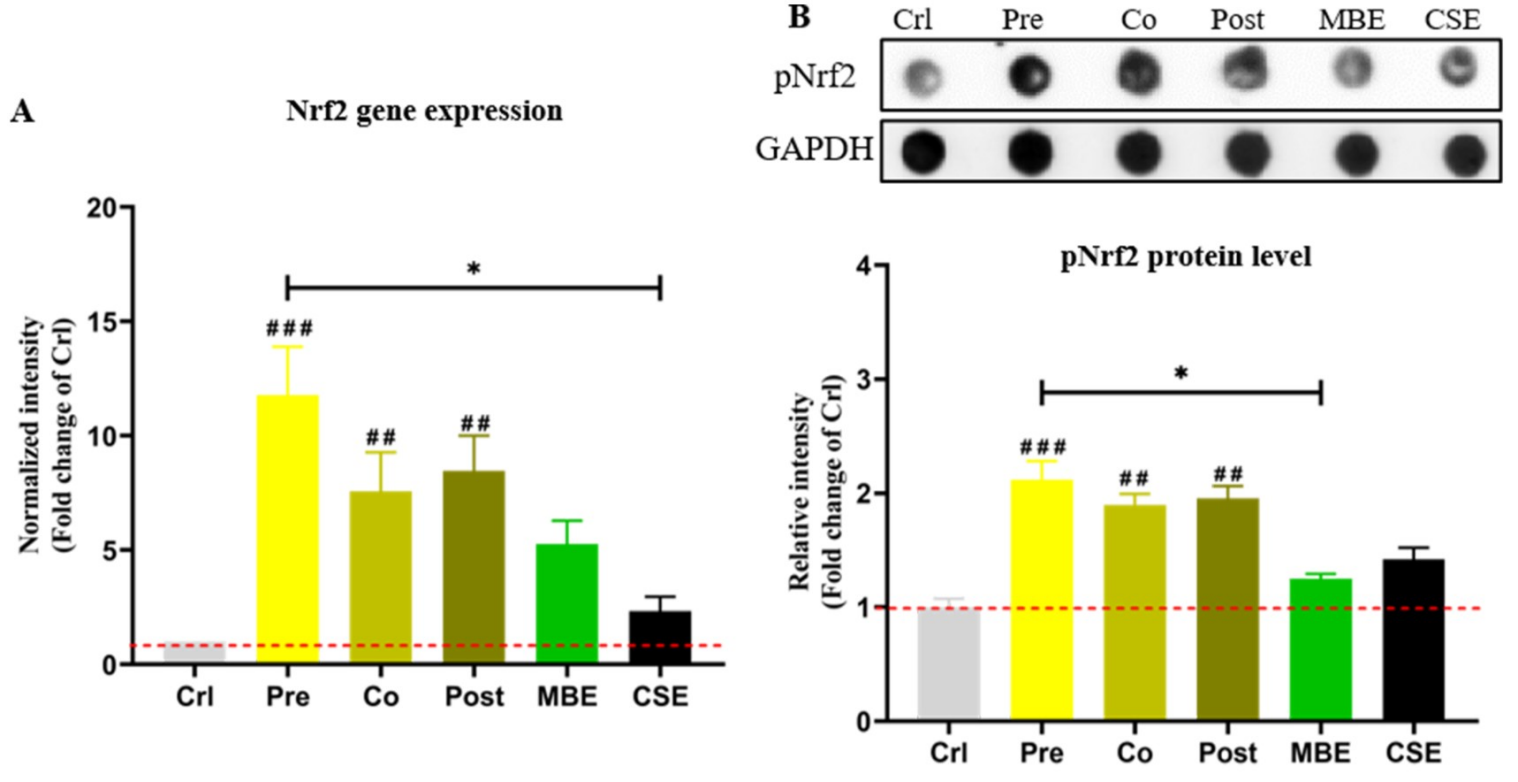
The gene and protein expression of Nrf2 in hOBs under different exposure strategies of CSE and MBE. (A) *Nrf2* gene expression on day 14 of culture. (B) Dot blot showing pNrf2 levels in hOBs on day 14 with different exposure strategies as described. GAPDH was used as housekeeper. Data are represented as mean ± SEM. Significance is represented as *p < 0.05, and ^##^*p* <0.001, and ^###^*p* <0.001 vs. Crl group (pooled samples of three rounds).

Nrf2 controls the basal and induced expression of an array of antioxidant response element-dependent genes to regulate the intracellular anti-oxidative system (Ma, 2013[29]). Phosphorylated Nrf2 (pNrf2) is the activated form of native Nrf2 (Chen et al., 2016[5]). *Nrf2* gene expression was determined by PCR and pNrf2 protein levels were determined by Dot blot in the present study. As shown in Figure 4(Fig. 4), CSE and MBE both increased *Nrf2* expression compared to the control group, and combined incubations with CSE and MBE showed higher *Nrf2* expression. Similar holds for the activation of pNrf2 protein. Among three combined incubation methods, pre-incubation with MBE showed the most pronounced activation effect on Nrf2.

### MBE and CSE exposure induce the expression of antioxidant enzymes in hOBs, and incubation with MBE could further increase their expressions

**Figure 5:**
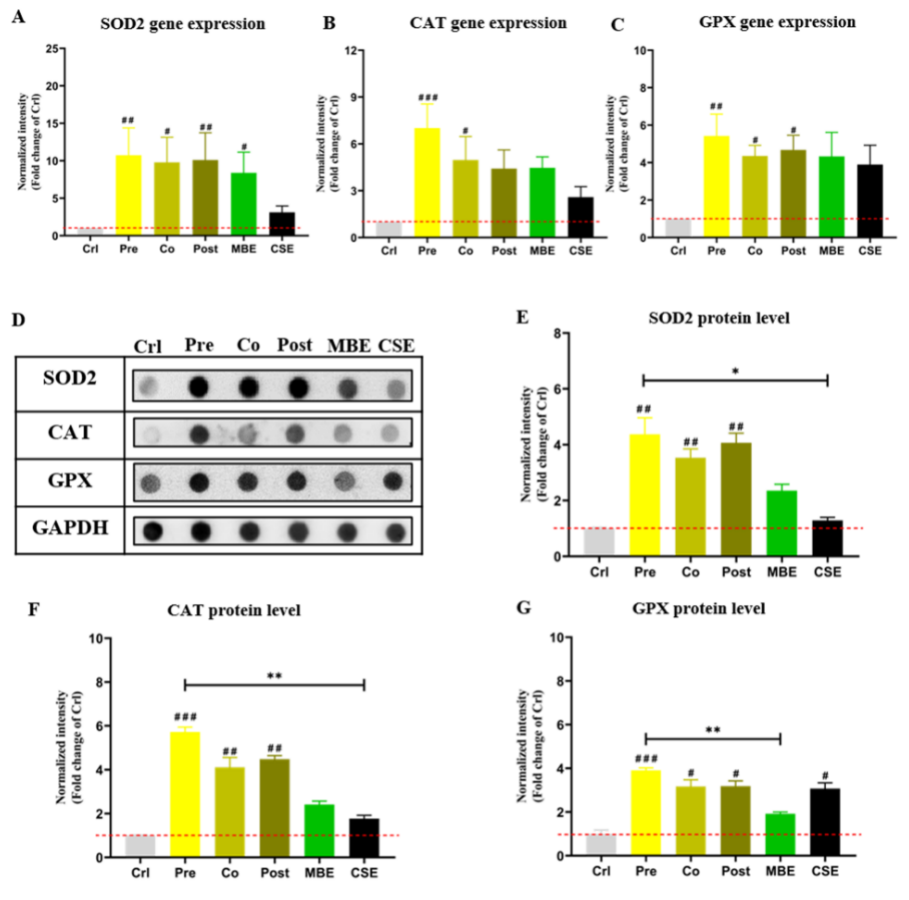
The gene and protein expression of antioxidative enzymes in hOBs under different exposure strategies of CSE and MBE. (A) *SOD2* gene expression measured by PCR on day 14. (B) *CAT* gene expression by PCR on day 14. (C) *GPX* gene expression by PCR on day 14. (D) Dot blot images of protein levels of the antioxidative enzymes (SOD2, CAT, and GPX) in hOBs on day 14 with different exposure strategies as described. GAPDH was used as housekeeper. (E) Protein levels of SOD2 measured by Dot blot on day 14. (F) Protein levels of CAT measured by Dot blot on day 14. (G) Protein levels of CAT measured by Dot blot on day 14. Data are represented as mean ± SEM. Significance is represented as *p < 0.05, and ^#^*p* < 0.05, ^##^*p* <0.001, and ^###^*p* <0.001 vs. Crl group (pooled samples of three rounds).

Nrf2-regulated anti-oxidative enzymes SOD2, CAT, and GPX were evaluated at the gene and protein level (Amaral et al., 2019[2]). As shown in Figure 5(Fig. 5), incubation with CSE or MBE increased the expression of antioxidant enzymes at the gene and protein level compared to the control group. Combined incubations of CSE and MBE showed higher expression of antioxidant enzymes at the gene and protein level. Specifically, pre-incubation with MBE showed the highest activation effect on the selected enzymes' expression among all groups.

### MBE prevents the detrimental effects of CSE-induced oxidative damage on hOBs

**Figure 6:**
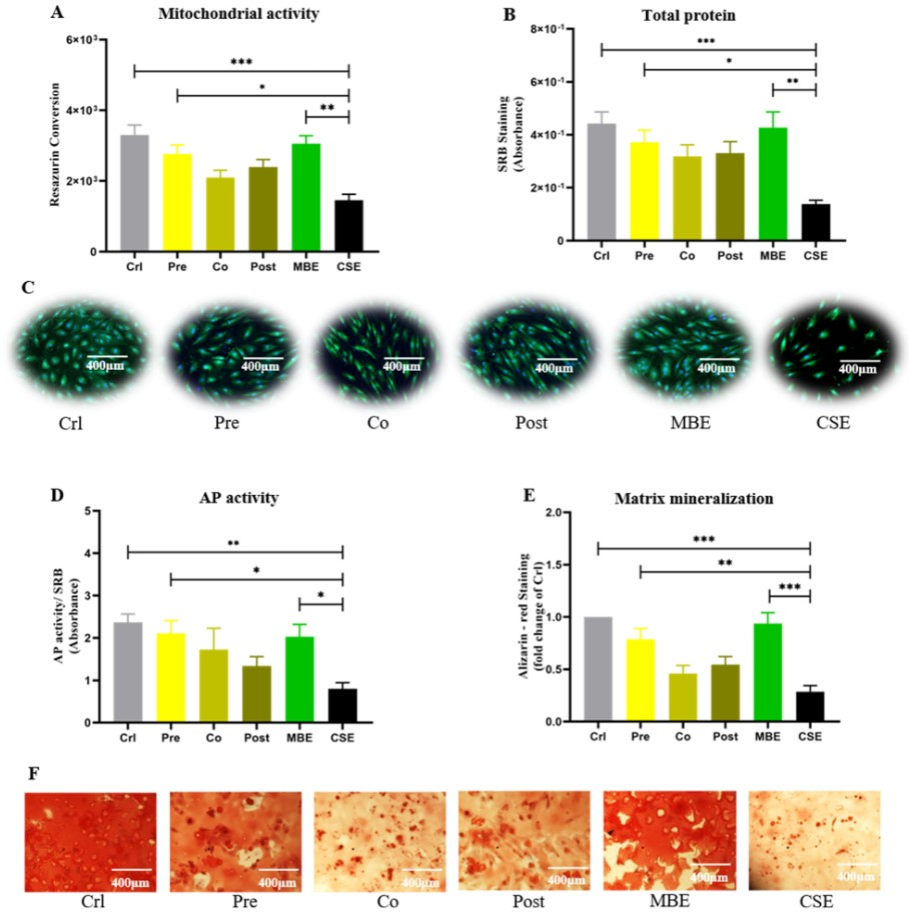
Effects of pre-/ co-/ post-incubation with MBE on CSE-affected hOBs. (A) Measurement of resazurin conversion in hOBs under different exposure strategies on day 14. (B) SRB staining of hOBs under different exposure strategies on day 14. (C) Representative live/dead staining images of hOBs under different exposure strategies on day 14. (D) AP activity of hOBs under different exposure strategies on day 14. (E) Alizarin-red staining of hOBs under different exposure strategies on day 21. Data are represented as mean ± SEM (N = 3, n = 3). Significance was represented as *p < 0.05, **p < 0.001, and ***p < 0. 0001. (F) Representative microscopy images of Alizarin-red staining of hOBs under different exposure strategies on day 21.

Cell viability, cell differentiation and matrix mineralization were evaluated to investigate the consequences of oxidative damage on hOBs. Resazurin conversion assay, SRB staining, and live/dead staining were used to determine hOB cell viability. Our results showed that CSE exposure significantly decreased hOB cell viability compared to the control and MBE groups; pre-incubation with MBE could significantly prevent the decreased cell viability caused by CSE. AP is an important marker for osteoblast activity during early osteoblast differentiation (Li et al., 2019[27]). As shown in Figure 6D(Fig. 6), CSE suppressed AP activity significantly compared to control and MBE groups, while pre-incubation with MBE significantly enhanced AP activity that was reduced by CSE. Alizarin-red staining was used to determine hOB mineralization (Takanche et al., 2020[48]). Similar to AP activity, CSE exposure decreased hOB matrix mineralization compared to control and MBE groups, and was significantly compensated by pre-incubation with MBE (Figure 6(Fig. 6)).

See also the Supplementary data.

## Discussion

MBE, which is recently more of international interest for its potential antioxidant benefits, is predominated with anthocyanins, indole alkaloids and flavonoids, coumarins, and caffeic as well as ferulic acids (Masoodi et al., 2019[33]; Quispe-Fuentes et al., 2018[41]). As a standardized, highly water-soluble powdered MBE, Delphinol® was demonstrated to prevent bone loss and improve bone metabolism in an osteoporotic mouse model (Moriwaki et al., 2014[35]; Nagaoka et al., 2019[36]). However, the specific effects of MBE on human bone cells have not been validated, and the underlying mechanisms of its antioxidative effects on preventing bone loss are unclear. Here, we tested the effects of different concentrations of Delphinol® on hOBs, focusing on its effects on cell viability and oxidative stress. 25 μg/mL of MBE, which was used to test on a mouse osteoblastic cell line mentioned above, was shown to promote bone metabolism. Interestingly, this concentration of MBE used in the only study to investigate the relationship between MBE and osteoblasts, significantly decreased the cell viability and increased oxidative stress of hOBs in our study. Our data suggest that the appropriate concentration of MBE differs between humans and animals. High concentrations of MBE showed prooxidative effect on hOBs, while low concentrations of MBE did not show cytotoxicity to hOBs or increased the oxidative stress. A bioavailability study showed the maximal concentration of delphinidin-3-O-glucoside (part of the proprietary MBE) could reach approximately 0.64 μg/mL in healthy humans (Schon et al., 2018[45]). As MBE is standardized to 25 % delphinidin glycosides (Alvarado et al., 2016[1]), 1.56 μg/mL MBE showed the highest cell viability in our study and is within the physiological concentration range of a healthy human. Therefore, 1.56 μg/mL MBE was selected to use for subsequent experiments.

CS is associated with a multitude of bone metabolism disorders and diseases, and one of the main mechanisms of CS-induced damage is the activation of oxidative stress. CS has been shown by our previous studies to induce oxidative stress in mesenchymal stem cells (Aspera-Werz et al., 2018[3]), chondrocytes (Chen et al., 2020[6]), and osteoblasts (Holzer et al., 2012[19]), thereby damaging the cells. The ROS level and nitrotyrosine are considered as important biomarkers of oxidative stress (Sun et al., 2019[47]). In our study, CSE could be used as a potent inducer of oxidative stress as it significantly increased both ROS and nitrotyrosine levels in hOBs. Incubation with MBE significantly decreased ROS levels and nitrotyrosine production in hOBs stimulated by CSE, indicating that MBE may exert an indirect protective effect by triggering the cellular antioxidant system. 

We investigated the response of the cellular antioxidant system of hOBs to CSE and MBE. Oxidative stress is able to induce cellular antioxidant responses, and Nrf2 is the master regulator of antioxidant defenses that regulate the transcription of antioxidant enzymes like SOD, CAT, and GPX (Done and Traustadottir, 2016[10]). We found that CSE and MBE up-regulated both gene expression and protein activation of Nrf2 in hOBs. The elevated Nrf2 levels following CSE exposure is likely due to the increased ROS levels induced by CSE, which cause conformational changes of Keap-1 protein and released Nrf2 for activation of physiological antioxidative defenses (Zhang et al., 2015[53]). However, hOBs exposed to MBE were not observed to activate ROS or nitrotyrosine production, suggesting MBE, as an antioxidant, can activate the expression of Nrf2. This result is consistent with another study which showed that delphinidin can activate Nrf2 as an epigenetic demethylating agent of the Nrf2 promoter (Kuo et al., 2019[24]). We exposed MBE and CSE to hOBs in three incubation modes including pre-incubation, co-incubation and post-incubation as described. All three incubation modes further enhanced Nrf2 expression in hOBs compared to MBE or CSE exposure alone, indicating that MBE counteracted oxidative stress induced by CSE by activating antioxidative defense system. Among the three incubation modes, pre-incubation with MBE showed the most pronounced effect on activating Nrf2. Similarly, Nrf-2-regulated antioxidative enzymes SOD2, CAT, and GPX showed similar patterns as Nrf2 in our study (Mahmoud et al., 2017[31]). CSE increased both the gene and protein expression of these enzymes via activating the cellular antioxidative response, and incubation with MBE, especially the pre-incubation method could further enhance the expression of SOD2, CAT, and GPX at both the gene and protein level. The up-regulated expression of antioxidative enzymes due to delphinidin in mouse models has been reported previously (Chen et al., 2018[7]; Intuyod et al., 2014[21]). MBE thus counteracted CSE-induced oxidative stress by activating the regulator of antioxidative defense system Nrf2 and its regulated functional antioxidant enzymes in hOBs (Table 3(Tab. 3)).

**Figure 7:**
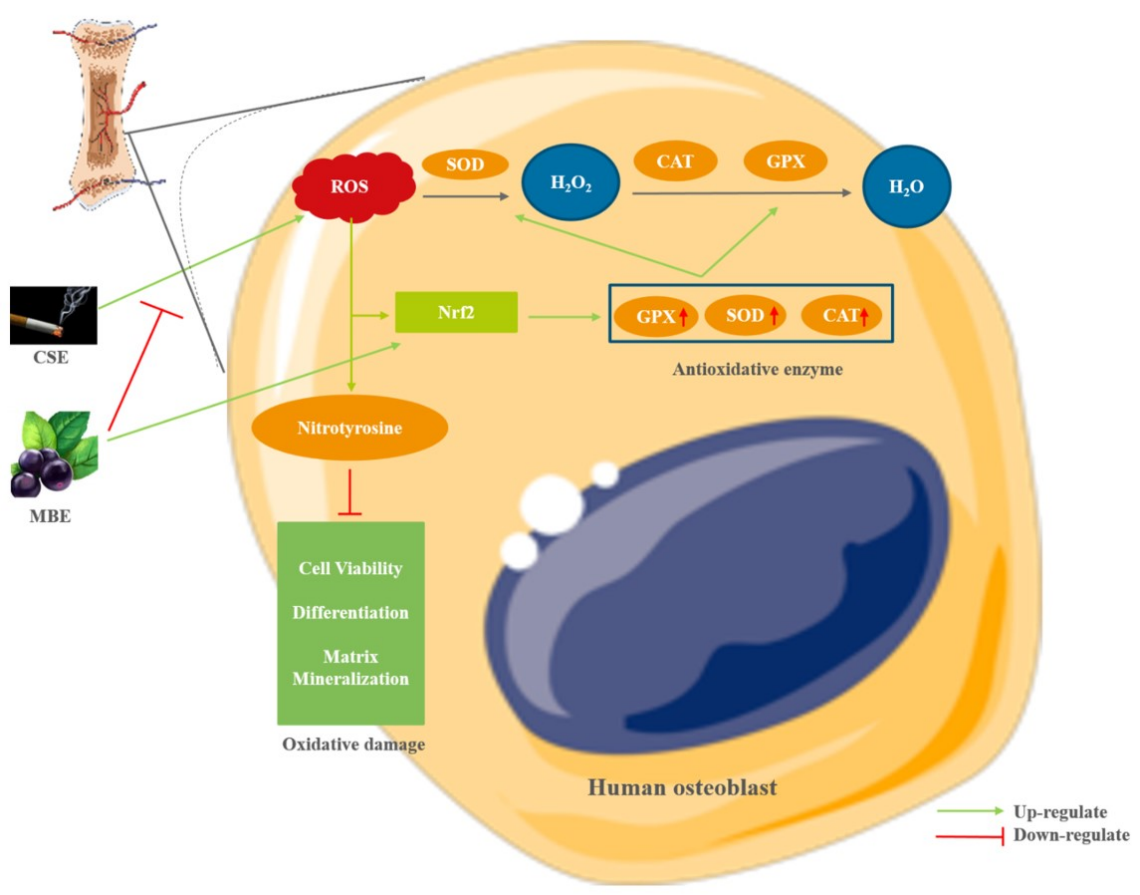
The effect of MBE and CSE on hOBs. CSE generated high level of oxidative stress by increasing ROS level, which caused oxidative damage during hOBs differentiation. MBE could counteract the negative effects of CSE on hOBs by activating the Nrf2-regulated antioxidative defense system, including the antioxidative enzymes SOD, CAT and GPX to remove ROS. (Graphical components were obtained from https://smart.servier.com/)

In addition to the oxidative status of hOBs exposed to CSE and MBE, cell damage due to oxidative stress was also evaluated. Similar to previous studies (Braun et al., 2011[4]; Marinucci et al., 2014[32]), CSE in our study significantly reduced hOBs cell viability, differentiation, and matrix mineralization compared to the control group, emphasizing the negative role of oxidative stress induced by CSE which can cause osteoporotic alterations during hOBs differentiation. It is the first time to test the effects of MBE on human bone cells *in vitro*, and MBE showed no obvious effect on hOBs in the absence of increased oxidative stress during osteogenic differentiation. Consistent with our results, an *in vivo* study reported that delphinidin had no effect on osteoblast differentiation and distribution in a fish model (Imangali et al., 2020[20]). Moreover, incubation with MBE enhanced hOB cell viability, differentiation, and matrix mineralization suppressed by CSE, suggesting MBE had a protective effect on CSE-impaired hOBs (Figure 7(Fig. 7)). Likewise, other *in vivo *studies found that Maqui berry or delphinidin had no direct effect on osteoblasts, but showed a positive effect under osteoporotic conditions (Moriwaki et al., 2014[35]; Nagaoka et al., 2019[36]). As demonstrated above, both MBE and CSE could activate the cellular antioxidant denfense system, and the pre-incubation method showed the most dramatic effect on activating antioxidative defense system. This suggests that pre-exposure of hOBs to MBE can activate the cellular antioxidant system to a greater extent, which may have implications for the clinical application of MBE. Therefore, our results suggest that MBE intake before the onset of osteoblastic damage induced by oxidative stress may be beneficial for bone maintenance. However, more *in vivo* studies are needed to validate the effect of different incubation methods of MBE on the skeletal system.

## Conclusion

In summary, MBE is tested for the first time in untreated and CSE-impaired hOBs* in vitro*. At a physiological concentration, MBE can effectively protect osteoblasts from oxidative stress-induced osteoporotic damage. One possible underlying mechanism is that MBE can activate the antioxidative defense system by increasing the expression and activation of Nrf2 and its regulated antioxidative enzymes. Interestingly, although both CSE and MBE could activate the cellular antioxidant system of hOBs, pre-incubation with MBE has the most pronounced activating effect.

## Acknowledgements

This research was partially funded by institutional funds. Sheng Zhu, Tao Chen and Weidong Weng are research scholars supported by China Scholarship Council. Maqui berry extract was a kindly gift by Anklam Extrakt GmbH, Anklam, Germany.

## Conflict of interest

The authors declare no conflict of interest. 

## Supplementary Material

Supplementary data

## Figures and Tables

**Table 1:**
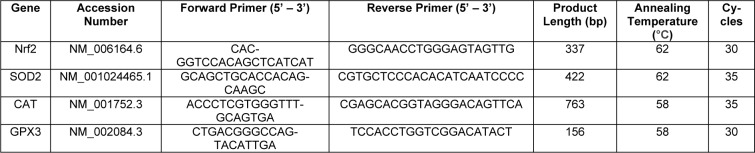
Primer sequences and PCR conditions for the investigated genes

**Table 2:**
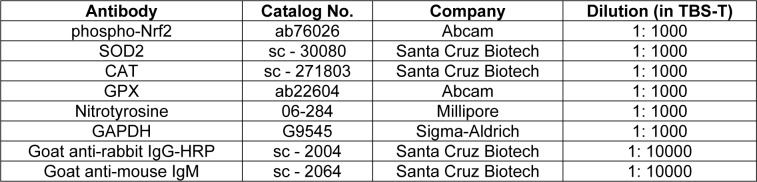
Information of antibodies used in the study

**Table 3:**
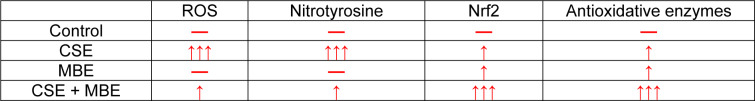
The overview of oxidative and antioxidative measurements in hOBs under different exposure strategies of CSE and MBE. Upward arrows in red color indicate increases compared to control group, and horizontal line in red indicates no significant difference compared to the control group.
