# Supplementary material for: Maqui berry extract prevents cigarette smoke induced oxidative stress in human osteoblasts in vitro
Source: EXCLI J. 2021 Feb 9;20:281–96. doi: 10.17179/excli2020-3244 (PMC7898044; doi:10.17179/excli2020-3244)
Supplement: Supplementary data [file EXCLI-20-281-s-001.pdf]

EXCLI Journal 2021;20:281-296 – ISSN 1611-2156
Received: December 03, 2020, accepted: February 04, 2021, published: February 09, 2021

Supplementary data to:

Original article:

MAQUI BERRY EXTRACT PREVENTS CIGARETTE SMOKE INDUCED OXIDATIVE STRESS IN HUMAN OSTEOBLASTS *IN VITRO*

Sheng Zhu, Romina H. Aspera-Werz, Tao Chen, Weidong Weng, Bianca Braun, Tina Histing, Andreas K. Nüssler*

Department of Traumatology, Eberhard Karls University Tübingen, BG Clinic, Siegfried Weller Institute, Schnarrenbergstraße 95, 72076 Tübingen, Germany

* **Corresponding author:** Andreas K. Nüssler, Department of Traumatology, Eberhard Karls University Tübingen, BG Clinic, Siegfried Weller Institute, Schnarrenbergstraße 95, 72076 Tübingen, Germany. Tel.: +49-7071-606-1065;
E-mail: andreas.nuessler@med.uni-tuebingen.de

https://orcid.org/0000-0002-6666-6791 (Andreas Nussler)

http://dx.doi.org/10.17179/excli2020-3244

This is an Open Access article distributed under the terms of the Creative Commons Attribution License (http://creativecommons.org/licenses/by/4.0/).

Raw data of Figure 2A: Resazurin conversion of hOBs exposed to different concentrations of MBE on day 1, day 3, and day 7

Day 1

Raw data						
	**25**	**6.25**	**3.12**	**1.56**	**0.156**	**Crl**
R1	956.6667	3979.667	4171.667	5835.667	5264.667	5119.667
	1076.667	2760.667	4660.667	5937.667	6238.667	5529.667
	1857.667	5686.667	4875.667	5723.667	5504.667	5621.667
R2	41.33333	2969.333	2423.333	1697.333	3485.333	3607.333
	210.3333	2216.333	4143.333	2401.333	4286.333	2862.333
	67.33333	3309.333	2747.333	4514.333	3978.333	1730.333
R3	721.3333	4133.333	1891.333	3996.333	1801.333	3567.333
	2041.333	1903.333	4560.333	4057.333	3029.333	2674.333
	2249.333	4601.333	4304.333	5083.333	3483.333	4505.333
Fold change of Crl						
	**25**	**6.25**	**3.12**	**1.56**	**0.156**	**Crl**
R1	0.176387	0.733759	0.76916	1.075963	0.970684	0.943949
	0.198513	0.509004	0.85932	1.09477	1.150267	1.019544
	0.342511	1.048491	0.898961	1.055313	1.014935	1.036507
R2	0.015122	1.086341	0.886585	0.620976	1.275122	1.319756
	0.076951	0.810854	1.515854	0.878537	1.568171	1.047195
	0.024634	1.210732	1.005122	1.651585	1.455488	0.633049
R3	0.120766	0.692003	0.316647	0.669066	0.301579	0.597243
	0.34176	0.318656	0.763491	0.679279	0.507171	0.447737
	0.376584	0.770355	0.720632	0.851052	0.58318	0.754283
AVG	0.2481	0.9299	0.9931	1.116	1.084	1.000
SEM	0.07453	0.09732	0.09859	0.09762	0.1079	0.07213

Day 3

Raw data						
	25	6.25	3.12	1.56	0.156	Crl
R1	1113.667	2305.667	4673.667	5025.667	5585.667	4272.667
	33.66667	4057.667	3651.667	5468.667	5047.667	3068.667
	1313.667	4231.667	4493.667	4409.667	4360.667	4420.667
R2	417.6667	1922.667	2262.667	3342.667	2846.667	2423.667
	1656.667	2314.667	2825.667	2849.667	2735.667	2742.667
	1680.667	2541.667	2710.667	2406.667	2417.667	2534.667
R3	964.6667	2957.667	2747.667	2541.667	2549.667	2050.667
	462.6667	2932.667	2830.667	2622.667	2782.667	2271.667
	659.6667	3099.667	3607.667	3020.667	2983.667	2373.667

EXCLI Journal 2021;20:281-296 – ISSN 1611-2156
Received: December 03, 2020, accepted: February 04, 2021, published: February 09, 2021

Fold change of Crl						
	**25**	**6.25**	**3.12**	**1.56**	**0.156**	**Crl**
R1	0.28405	0.58808	1.192059	1.28184	1.424673	1.089781
	0.008587	1.034943	0.931389	1.394831	1.287451	0.78269
	0.335062	1.079323	1.146149	1.124724	1.112226	1.127529
R2	0.162706	0.748994	0.881444	1.302169	1.108947	0.944163
	0.645371	0.901701	1.100766	1.110116	1.065706	1.068433
	0.65472	0.990131	1.055967	0.937541	0.941826	0.987404
R3	0.432198	1.325119	1.231033	1.13874	1.142324	0.918757
	0.207288	1.313919	1.26822	1.17503	1.246714	1.017772
	0.29555	1.38874	1.616338	1.353345	1.336768	1.063471
AVG	0.3362	1.041	1.158	1.202	1.185	1.000
SEM	0.07117	0.09053	0.07172	0.04796	0.05015	0.03544

Day 7

Raw data						
	**25**	**6.25**	**3.12**	**1.56**	**0.156**	**Crl**
R1	4021	6379	7629	6187	6358	8027
	4368	5650	6236	6775	7053	8228
	4516	5117	5200	6369	6256	6238
R2	164.6667	834.6667	1826.667	998.6667	1345.667	3119.667
	46.66667	2272.667	2147.667	2779.667	1389.667	1523.667
	77.66667	3287.667	677.6667	3045.667	871.6667	3464.667
R3	680.6667	3440.667	3437.667	3617.667	2178.667	2039.667
	389.6667	3288.667	3773.667	3569.667	1229.667	3395.667
	116.6667	3532.667	1366.667	3507.667	3993.667	2771.667
Fold change of Crl						
	**25**	**6.25**	**3.12**	**1.56**	**0.156**	**Crl**
R1	0.5363	0.850798	1.017517	0.82519	0.847997	1.0706
	0.582581	0.753568	0.831725	0.903614	0.940693	1.097408
	0.602321	0.682479	0.693549	0.849464	0.834393	0.831992
R2	0.060927	0.308831	0.675876	0.369512	0.497903	1.154292
	0.017267	0.840898	0.794647	1.02849	0.514184	0.563764
	0.028737	1.216453	0.25074	1.126912	0.322521	1.281944
R3	0.248812	1.257707	1.25661	1.322408	0.796393	0.745583
	0.142439	1.202145	1.379432	1.304862	0.449494	1.241257
	0.042647	1.291337	0.499574	1.282198	1.459851	1.013159
AVG	0.2513	0.9338	0.8222	1.001	0.7404	1.000
SEM	0.07988	0.1149	0.1023	0.1183	0.1110	0.08417

EXCLI Journal 2021;20:281-296 – ISSN 1611-2156
Received: December 03, 2020, accepted: February 04, 2021, published: February 09, 2021

Raw data of Figure 2B: SRB staining of hOBs exposed to different concentrations of MBE on day 1, day 3, and day 7

Day 1

Raw data						
	**25**	**6.25**	**3.12**	**1.56**	**0.156**	**Crl**
R1	0.0295	0.0535	0.1575	0.2085	0.1765	0.1635
	0.0535	0.0845	0.1465	0.1745	0.2225	0.1395
	0.0695	0.1615	0.1475	0.1795	0.2275	0.1995
R2	0.033	0.086	0.069	0.158	0.136	0.132
	0.045	0.157	0.106	0.175	0.202	0.138
	0.089	0.117	0.149	0.16	0.163	0.154
R3	0.17	0.178	0.207	0.295	0.233	0.221
	0.169	0.243	0.215	0.155	0.151	0.213
	0.157	0.253	0.241	0.209	0.181	0.227
Fold change of Crl						
	**25**	**6.25**	**3.12**	**1.56**	**0.156**	**Crl**
R1	0.176119	0.319403	0.940299	1.244776	1.053731	0.976119
	0.319403	0.504478	0.874627	1.041791	1.328358	0.832836
	0.414925	0.964179	0.880597	1.071642	1.358209	1.191045
R2	0.14248	0.562005	0.427441	1.131926	0.957784	0.926121
	0.237467	1.124011	0.720317	1.266491	1.480211	0.973615
	0.585752	0.807388	1.060686	1.147757	1.171504	1.100264
R3	0.75487	0.793831	0.935065	1.363636	1.061688	1.003247
	0.75	1.11039	0.974026	0.681818	0.662338	0.964286
	0.691558	1.159091	1.100649	0.944805	0.808442	1.032468
AVG	0.4525	0.8161	0.8793	1.099	1.098	1.000
SEM	0.03407	0.08889	0.06715	0.06741	0.1007	0.08272

Day 3

Raw data						
	**25**	**6.25**	**3.12**	**1.56**	**0.156**	**Crl**
R1	0.0985	0.0875	0.2185	0.2675	0.2355	0.2735
	0.0375	0.2195	0.2185	0.3715	0.2825	0.3325
	0.2765	0.2975	0.3025	0.3355	0.3725	0.2965
R2	0.016	0.126	0.121	0.189	0.122	0.146
	0.013	0.126	0.122	0.124	0.123	0.137
	0.05	0.133	0.139	0.131	0.134	0.131
R3	0.078	0.111	0.078	0.201	0.168	0.239
	0.072	0.18	0.17	0.146	0.16	0.206
	0.087	0.15	0.053	0.23	0.197	0.181

EXCLI Journal 2021;20:281-296 – ISSN 1611-2156
Received: December 03, 2020, accepted: February 04, 2021, published: February 09, 2021

Fold change of Crl						
	**25**	**6.25**	**3.12**	**1.56**	**0.156**	**Crl**
R1	0.327424	0.290859	0.726316	0.889197	0.782825	0.909141
	0.124654	0.72964	0.726316	1.234903	0.939058	1.105263
	0.919114	0.98892	1.00554	1.115235	1.238227	0.985596
R2	0.115942	0.913043	0.876812	1.369565	0.884058	1.057971
	0.094203	0.913043	0.884058	0.898551	0.891304	0.992754
	0.362319	0.963768	1.007246	0.949275	0.971014	0.949275
R3	0.373802	0.531949	0.373802	0.963259	0.805112	1.145367
	0.345048	0.86262	0.814696	0.699681	0.766773	0.98722
	0.416933	0.71885	0.253994	1.102236	0.944089	0.867412
AVG	0.3422	0.7681	0.7410	1.025	0.9136	1.000
SEM	0.08334	0.07687	0.08801	0.06736	0.04744	0.02991

Day 7

Raw data						
	**25**	**6.25**	**3.12**	**1.56**	**0.156**	**Crl**
R1	0.0535	0.2535	0.2965	0.1825	0.1815	0.2425
	0.1585	0.2145	0.2195	0.2665	0.2765	0.2415
	0.1765	0.2315	0.2395	0.2115	0.2565	0.2725
R2	0.036	0.139	0.139	0.248	0.165	0.15
	0.114	0.153	0.153	0.173	0.177	0.171
	0.027	0.197	0.185	0.199	0.233	0.209
R3	0.122	0.139	0.106	0.189	0.15	0.201
	0.121	0.122	0.189	0.224	0.175	0.195
	0.118	0.095	0.182	0.278	0.25	0.166
Fold change of Crl						
	**25**	**6.25**	**3.12**	**1.56**	**0.156**	**Crl**
R1	0.212161	1.005288	1.17581	0.723728	0.719762	0.961666
	0.628553	0.850628	0.870456	1.056841	1.096497	0.9577
	0.699934	0.918044	0.949769	0.838731	1.017184	1.080635
R2	0.203774	0.786792	0.786792	1.403774	0.933962	0.849057
	0.645283	0.866038	0.866038	0.979245	1.001887	0.967925
	0.15283	1.115094	1.04717	1.126415	1.318868	1.183019
R3	0.651246	0.741993	0.565836	1.008897	0.800712	1.072954
	0.645907	0.651246	1.008897	1.19573	0.934164	1.040925
	0.629893	0.507117	0.97153	1.483986	1.33452	0.886121
AVG	0.4966	0.8269	0.9158	1.091	1.018	1.000
SEM	0.07725	0.06092	0.05791	0.08194	0.06946	0.03477

EXCLI Journal 2021;20:281-296 – ISSN 1611-2156
Received: December 03, 2020, accepted: February 04, 2021, published: February 09, 2021

Raw data of Figure 2D: ROS level measurements of hOBs exposed to different concentrations of MBE on day 1

Raw data							
	**Crl**	**0.156**	**1.56**	**3.12**	**6.25**	**25**	**H_2O_2**
R1	202	188	264	277	580	560	2289
	254	204	274	294	374	532	2389
	195	202	280	329	394	549	2419
R2	190	179	161	288	831	600	1887
	248	199	274	317	421	582	2113
	200	208	178	331	417	592	2009
R3	202	182	272	491	830	602	1791
	244	205	275	306	412	567	1681
	193	204	86	331	424	595	1909
Fold change of Crl							
	**Crl**	**0.156**	**1.56**	**3.12**	**6.25**	**25**	**H_2O_2**
R1	1	0.930693	1.306931	1.371287	2.871287	2.772277	11.33168
	1	0.80315	1.07874	1.15748	1.472441	2.094488	9.405512
	1	1.035897	1.435897	1.687179	2.020513	2.815385	12.40513
R2	1	0.942105	0.847368	1.515789	4.373684	3.157895	9.931579
	1	0.802419	1.104839	1.278226	1.697581	2.346774	8.520161
	1	1.04	0.89	1.655	2.085	2.96	10.045
R3	1	0.90099	1.346535	2.430693	4.108911	2.980198	8.866337
	1	0.840164	1.127049	1.254098	1.688525	2.32377	6.889344
	1	1.056995	0.445596	1.715026	2.196891	3.082902	9.891192
AVG	1.000	0.9280	1.065	1.563	2.502	2.726	1.000
SEM	0.000	0.03351	0.1016	0.1280	0.3553	0.1263	0.000

EXCLI Journal 2021;20:281-296 – ISSN 1611-2156
Received: December 03, 2020, accepted: February 04, 2021, published: February 09, 2021

Raw data of Figure 2E: ROS level measurements of hOBs exposed to different concentrations of MBE on day 3

Raw data							
	**Crl**	**0.156**	**1.56**	**3.12**	**6.25**	**25**	**H_2O_2**
R1	183	191	172	224	214	269	1789
	189	178	213	185	270	298	1671
	139	231	210	212	240	255	1983
R2	185	218	181	232	221	351	1567
	191	202	228	184	261	301	1987
	156	224	212	212	244	259	1789
R3	189	216	280	223	221	250	1516
	188	204	221	299	267	301	1489
	165	136	212	214	251	340	1781
Fold change of Crl							
	**Crl**	**0.156**	**1.56**	**3.12**	**6.25**	**25**	**H_2O_2**
R1	1	1.043716	0.939891	1.224044	1.169399	1.469945	9.775956
	1	0.941799	1.126984	0.978836	1.428571	1.57672	8.84127
	1	1.661871	1.510791	1.52518	1.726619	1.834532	14.26619
R2	1	1.178378	0.978378	1.254054	1.194595	1.897297	8.47027
	1	1.057592	1.193717	0.963351	1.366492	1.575916	10.40314
	1	1.435897	1.358974	1.358974	1.564103	1.660256	11.46795
R3	1	1.142857	1.481481	1.179894	1.169312	1.322751	8.021164
	1	1.085106	1.175532	1.590426	1.420213	1.601064	7.920213
	1	0.824242	1.284848	1.29697	1.521212	2.060606	10.79394
AVG	1.000	1.152	1.228	1.264	1.396	1.667	1.000
SEM	0.000	0.08483	0.06709	0.07123	0.06445	0.07585	0.000

EXCLI Journal 2021;20:281-296 – ISSN 1611-2156
Received: December 03, 2020, accepted: February 04, 2021, published: February 09, 2021

Raw data of Figure 3A: The ROS level of hOBs under different exposure strategies of CSE and MBE

Raw data						
	**H_2O_2**	**CSE**	**Maqui**	**Co-**	**Pre-**	**CrI**
R1	7352	4534	2469	3221	3185	2203
	5813	3485	1861	2506	2417	1446
	4463	2789	1401	2085	1972	1302
	4674	3188	1515	2460	2310	1418
	5999	3357	1516	2462	1516	1372
	5533	2965	1385	2551	2154	1284
R2	1450	1350	1237	1280	1277	1219
	1830	1640	1365	1486	1446	1309
	1697	1556	1320	1446	1412	1313
	1938	1784	1425	1629	1604	1384
	1783	1581	1311	1451	1296	1289
	1874	1603	1344	1546	1484	1296
R3	2622	1988	1517	1596	1659	1408
	2235	1784	1432	1476	1527	1400
	2571	1973	1491	1548	1582	1423
	2512	1838	1414	1446	1472	1388
	2134	1614	1366	1408	1427	1331
	2694	2156	1438	1539	1552	1377
Fold change of CrI						
	**H_2O_2**	**CSE**	**Maqui**	**Co-**	**Pre-**	**CrI**
R1	3.3372674	2.0581026	1.1207444	1.4620971	1.4457558	1
	4.0200553	2.4100968	1.2869986	1.7330567	1.6715076	1
	3.4278034	2.1420891	1.0760369	1.6013825	1.5145929	1
	3.2961918	2.248237	1.0684062	1.7348378	1.629055	1
	4.372449	2.446793	1.1049563	1.7944606	1.1049563	1
	4.30919	2.30919	1.0786604	1.9867601	1.6775701	1
R2	2.946903	2.106195	1.159292	1.517699	1.49115	1
	3.494565	2.586957	1.271739	1.847826	1.657609	1
	2.806122	2.142857	1.035714	1.627551	1.469388	1
	2.952941	2.411765	1.147059	1.864706	1.776471	1
	3.617834	2.547771	1.121019	1.859873	1.038217	1
	3.953846	2.569231	1.246154	2.276923	1.961538	1
R3	5.401003	3.102757	1.39599	1.684211	1.912281	1
	4.118492	2.43447	1.120287	1.287253	1.475763	1
	4.947589	2.893082	1.234801	1.431866	1.549266	1
	5.394191	2.761411	1.103734	1.228216	1.329876	1
	5.039419	2.423237	1.176349	1.390041	1.485477	1
	6.379501	4.180055	1.252078	1.66482	1.714681	1
AVG	4.101	2.543	1.167	1.666	1.550	1.000
SEM	0.2351	0.1161	0.02240	0.06124	0.05623	0.000

EXCLI Journal 2021;20:281-296 – ISSN 1611-2156
Received: December 03, 2020, accepted: February 04, 2021, published: February 09, 2021

Raw data of Figure 3B: The Nitrotyrosine level of hOBs under different exposure strategies of CSE and MBE (Area under curve). On Day 4 and Day 7, Pre-incubation group had the same exposure condition as MBE group and Post-incubation group had the same exposure condition as CSE group, so only 4 groups are shown on Day 4 and Day 7.

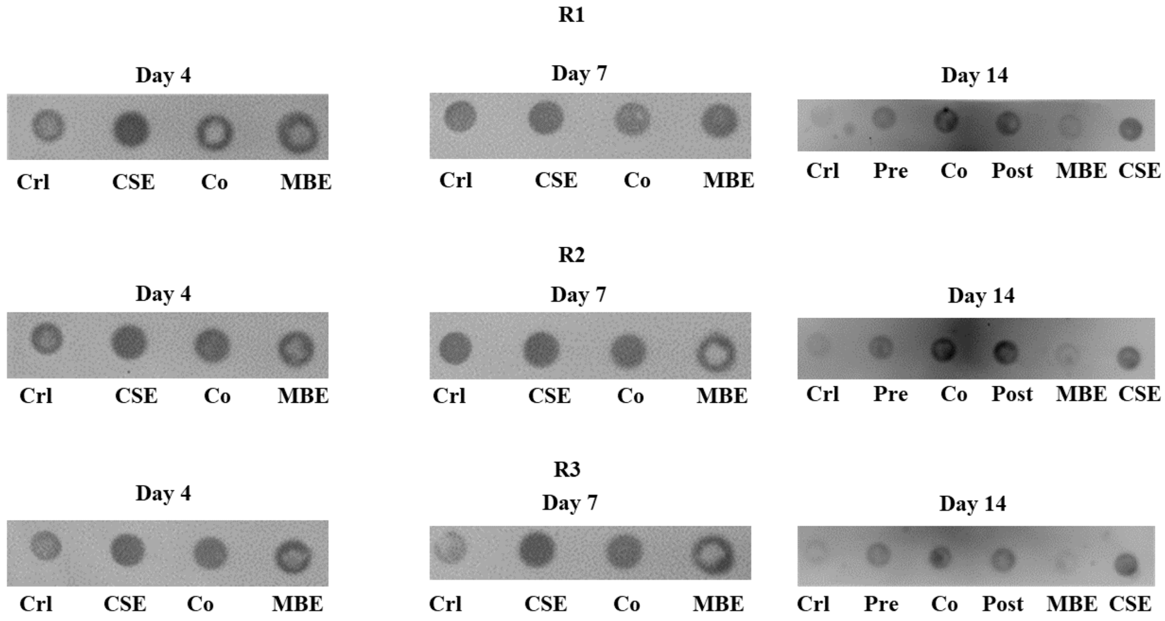

EXCLI Journal 2021;20:281-296 – ISSN 1611-2156
Received: December 03, 2020, accepted: February 04, 2021, published: February 09, 2021

Raw data of Figure 4A, 5A, 5B and 5C: PCR measurements of Nrf2, SOD2, CAT and GPX

Nrf2

Raw data						
	Crl	Pre	Co	Post	MBE	CSE
R1	3.504	44.0391	45.70335	39.61333	25.8243	9.114838
	3.041	50.16783	32.42128	35.31589	22.69755	7.098545
R2	1.571	19.03933	5.633157	14.42995	6.560413	10.03068
	13.461	74.98459	55.97962	59.48927	39.65516	15.36325
R3	2.491	51.53199	32.07846	32.41654	20.83776	6.490372
	4.267	67.07204	48.11404	51.69511	34.40169	7.875191
Fold change of Crl						
	Crl	Pre	Co	Post	MBE	CSE
R1	1	12.56824	13.04319	11.30518	7.369949	2.601267
	1	16.49715	10.66139	11.61325	7.463845	2.33428
R2	1	12.11924	3.585714	9.185199	4.175947	6.384903
	1	5.570506	4.158652	4.419379	2.94593	1.141316
R3	1	20.68727	12.87775	13.01346	8.36522	2.605529
	1	15.71878	11.27585	12.1151	8.062265	1.845604
AVG	1.000	13.86	9.267	10.28	6.397	2.819
SEM	0.000	2.085	1.748	1.281	0.9233	0.7485

SOD2

Raw data						
	**Crl**	**Pre**	**Co**	**Post**	**MBE**	**CSE**
R1	0.151	0.305864	0.276426	0.325596	0.226194	0.137802
	0.266	0.521629	0.479857	0.570489	0.39461	0.241919
R2	0.164	0.286896	0.22497	0.289419	0.240946	0.181694
	0.166	0.286896	0.227364	0.29081	0.242175	0.181694
R3	0.076	0.949601	0.890308	0.749984	0.808889	0.279687
	0.05	0.897438	0.812526	0.655366	0.720378	0.234773
	0.054	1.081194	1.02194	1.146543	0.891253	0.325621
	0.024	0.669819	0.600719	0.690152	0.472057	0.157196
Fold change of Crl						
	**Crl**	**Pre**	**Co**	**Post**	**MBE**	**CSE**
R1	1	2.02559	1.830638	2.156265	1.497972	0.912595
	1	1.96101	1.803974	2.144694	1.483495	0.909469
R2	1	1.749364	1.371771	1.764748	1.46918	1.107892
	1	1.728288	1.369661	1.751868	1.458885	1.094544
R3	1	12.49475	11.71458	9.86821	10.64327	3.680087
	1	17.94877	16.25052	13.10733	14.40756	4.695469
	1	20.02211	18.92481	21.23228	16.50468	6.03001
	1	27.90911	25.02995	28.75634	19.66902	6.549839
AVG	1.000	10.73	9.787	10.10	8.392	3.122
SEM	0.000	3.662	3.354	3.651	2.756	0.8550

EXCLI Journal 2021;20:281-296 – ISSN 1611-2156
Received: December 03, 2020, accepted: February 04, 2021, published: February 09, 2021

CAT

Raw data						
	**Crl**	**Pre**	**Co**	**Post**	**Maqui**	**CSE**
	0.068	0.238	0.213	0.171	0.166	0.154
	0.074	0.549	0.483	0.38	0.364	0.328
	0.032	0.09	0.037	0.035	0.079	0.035
	0.046	0.343	0.077	0.13	0.291	0.057
	0.278	0.856	0.763	0.697	0.591	0.338
	0.17	0.605	0.515	0.459	0.351	0.304
Fold change of Crl						
	**Crl**	**Pre**	**Co**	**Post**	**Maqui**	**CSE**
	1	3.5	3.132353	2.514706	2.441176	2.264706
	1	7.418919	6.527027	5.135135	4.918919	4.432432
	1	2.8125	1.15625	1.09375	2.46875	1.09375
	1	7.456522	1.673913	2.826087	6.326087	1.23913
	1	7.494382	6.449438	5.707865	4.516854	1.674157
	1	13.42857	10.85714	9.257143	6.171429	4.828571
AVG	1.000	7.018	4.966	4.422	4.474	2.589
SEM	0.000	1.546	1.506	1.194	0.6992	0.6687

GPX

Raw data						
	**Crl**	**Pre**	**Co**	**Post**	**MBE**	**CSE**
	0.019	0.199167	0.108895	0.136361	0.199149	0.159238
	0.026	0.219321	0.175908	0.246284	0.178251	0.127594
	0.154	0.505031	0.464301	0.574663	0.366336	0.4328
	0.053	0.288081	0.26805	0.354816	0.231111	0.280707
	0.026	0.170715	0.147188	0.126621	0.10695	0.08064
	0.587	1.238868	1.450341	1.049143	0.925673	0.782918
Fold change of Crl						
	**Crl**	**Pre**	**Co**	**Post**	**MBE**	**CSE**
	1	10.48249	5.731328	7.17688	10.48152	8.380928
	1	8.435428	6.76568	9.472467	6.855789	4.907472
	1	3.279424	3.014939	3.731578	2.378802	2.810388
	1	5.435495	5.057544	6.694644	4.360585	5.296366
	1	6.565954	5.66108	4.870026	4.113474	3.101522
	1	2.110508	2.470768	1.787296	1.576956	1.333762
AVG	1.000	5.419	4.355	4.676	4.330	3.896
SEM	0.000	1.173	0.5697	0.7829	1.288	1.032

EXCLI Journal 2021;20:281-296 – ISSN 1611-2156
Received: December 03, 2020, accepted: February 04, 2021, published: February 09, 2021

Raw data of Figure 5B, 6E, 6F and 6G: Protein level measurements of Nrf2, SOD2, CAT and GPX

1

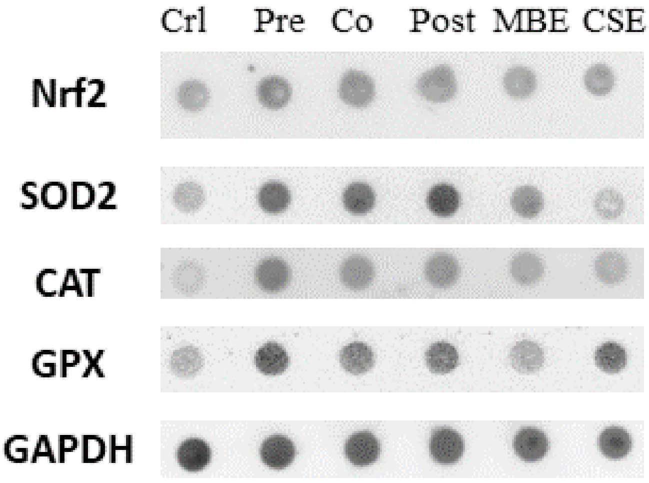

2

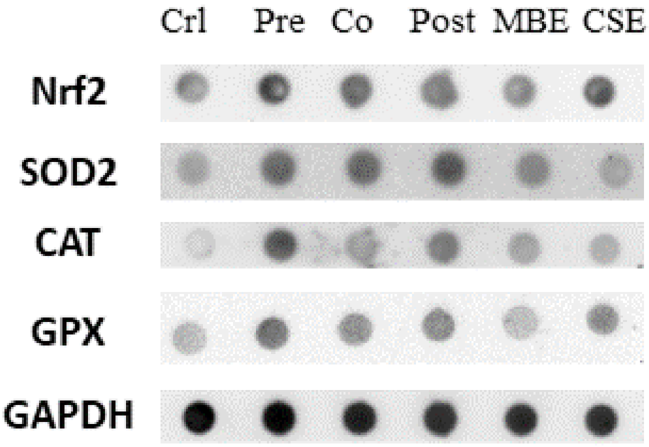

3

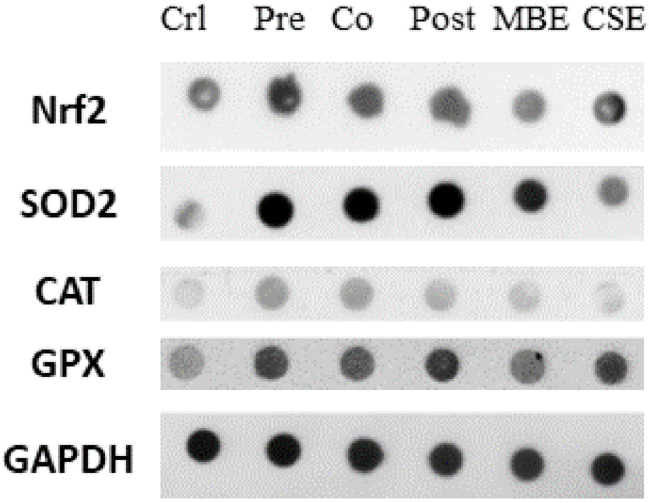

EXCLI Journal 2021;20:281-296 – ISSN 1611-2156
Received: December 03, 2020, accepted: February 04, 2021, published: February 09, 2021

Raw data of Figure 6A: Resazurin conversion in hOBs under different exposure strategies on day 14

Raw data						
	**CSE**	**MBE**	**Post**	**Co**	**Pre**	**Crl**
R1	2196	2738	1865	1168	2852	4352
	1969	3698	2714	2388	3450	3848
	2204	3901	2854	2706	4121	4215
R2	1153.5	2118.5	1438.5	1626.5	1823.5	2187.5
	990.5	1985.5	1761.5	1567.5	2702.5	2010.5
	1151.5	2892.5	1871.5	1766.5	1640.5	3718.5
R3	1142	3122	2634	1901	2380	3279
	1109	3525	3121	2700	3039	3317
	1162	3510	3240	3008	2879	2819
AVG	1453.1	3054.5	2388.8	2092.4	2765.3	3305.2
SEM	169.83	226.98	219.90	209.54	255.68	277.68

Raw data of Figure 6B: SRB staining of hOBs under different exposure strategies on day 14

Raw data						
	**CSE**	**MBE**	**Post**	**Co**	**Pre**	**Crl**
R1	0.184	0.283	0.208	0.243	0.262	0.377
	0.101	0.155	0.145	0.116	0.219	0.301
	0.071	0.352	0.163	0.196	0.172	0.292
R2	0.121	0.708	0.438	0.455	0.585	0.549
	0.168	0.591	0.508	0.297	0.338	0.426
	0.188	0.391	0.32	0.249	0.397	0.69
R3	0.182	0.555	0.423	0.369	0.445	0.436
	0.124	0.298	0.399	0.487	0.526	0.369
	0.103	0.512	0.369	0.452	0.398	0.541
AVG	0.1380	0.4272	0.3303	0.3182	0.3713	0.4423
SEM	0.0144	0.0587	0.0434	0.0432	0.0460	0.0433

EXCLI Journal 2021;20:281-296 – ISSN 1611-2156
Received: December 03, 2020, accepted: February 04, 2021, published: February 09, 2021

Raw data of Figure 6D: AP activity of hOBs under different exposure strategies on day 14

Raw data						
	**CSE**	**MBE**	**Post**	**Co**	**Pre**	**Crl**
R1	0.535326	1.966431	0.944712	0.829218	1.456107	1.754642
	0.688119	2.022581	1.975862	1.030172	1.125571	1.931894
	1.626761	1.03267	1.119632	1.461735	2.688953	2.738014
R2	1.259082	1.544846	1.632239	2.466063	1.87594	2.119977
	0.860017	1.790245	2.45139	3.475612	3.911572	3.024688
	0.447208	2.578921	1.779948	4.714471	3.004216	2.907288
R3	1.049451	1.356757	0.669031	0.823848	1.829213	2.238532
	0.41129	4.030201	1.077694	0.406571	1.671103	3.105691
	0.291262	1.96875	0.387534	0.325221	1.417085	1.512015
AVG	0.7965	2.032	1.338	1.726	2.109	2.370
SEM	0.1480	0.2899	0.2220	0.5057	0.3024	0.1965

Raw data of Figure 6E: Alizarin-red staining of hOBs under different exposure strategies on day 21

Raw data						
	**MBE**	**Post**	**Co**	**Pre**	**CSE**	**Crl**
R1	1.451	0.858	0.661	0.998	0.236	2.274
	1.243	0.35	0.414	0.536	0.569	1.78
	1.172	0.904	0.212	1.282	0.291	1.034
R2	1.749	0.703	0.877	0.862	0.558	1.184
	1.276	0.554	0.657	1.573	0.086	1.572
	1.162	0.668	0.472	1.062	0.081	1.205
R3	1.362	1.132	1.155	1.555	1	2.893
	2.091	1.652	1.228	1.782	0.981	1.982
	2.109	1.293	1.521	1.891	0.789	1.789
Fold change of Crl						
	**MBE**	**Post**	**Co**	**Pre**	**CSE**	**Crl**
R1	0.6380827	0.3773087	0.2906772	0.4388742	0.1037819	1
	0.6983146	0.1966292	0.2325843	0.3011236	0.3196629	1
	1.1334623	0.8742747	0.205029	1.2398453	0.2814313	1
R2	1.477196	0.59375	0.7407095	0.7280405	0.4712838	1
	0.8117048	0.3524173	0.4179389	1.0006361	0.0547074	1
	0.9643154	0.5543569	0.3917012	0.8813278	0.0672199	1
R3	0.4707916	0.3912893	0.3992395	0.5375043	0.3456619	1
	1.054995	0.8335015	0.6195762	0.8990918	0.4949546	1
	1.1788709	0.7227501	0.8501956	1.0570151	0.4410285	1
AVG	0.9364	0.5440	0.4609	0.7871	0.2866	1.000
SEM	0.1043	0.07781	0.07565	0.1033	0.05789	0.000
